# Pigment Dispersing Factor Regulates Ecdysone Biosynthesis via *Bombyx* Neuropeptide G Protein Coupled Receptor-B2 in the Prothoracic Glands of *Bombyx mori*

**DOI:** 10.1371/journal.pone.0103239

**Published:** 2014-07-29

**Authors:** Masatoshi Iga, Takayoshi Nakaoka, Yutaka Suzuki, Hiroshi Kataoka

**Affiliations:** Department of Integrated Biosciences, Graduate School of Frontier Sciences, The University of Tokyo, Kashiwa, Japan; U. Kentucky, United States of America

## Abstract

Ecdysone is the key hormone regulating insect growth and development. Ecdysone synthesis occurs in the prothoracic glands (PGs) and is regulated by several neuropeptides. Four prothoracicotropic and three prothoracicostatic factors have been identified to date, suggesting that ecdysone biosynthesis is intricately regulated. Here, we demonstrate that the neuropeptide pigment dispersing factor (PDF) stimulates ecdysone biosynthesis and that this novel signaling pathway partially overlaps with the prothoracicotropic hormone (PTTH) signaling pathway. We performed transcriptome analysis and focused on receptors predominantly expressed in the PGs. From this screen, we identified a candidate orphan G protein coupled receptor (GPCR), *Bombyx* neuropeptide GPCR-B2 (BNGR-B2). *BNGR-B2* was predominantly expressed in ecdysteroidogenic tissues, and the expression pattern in the PGs corresponded to the ecdysteroid titer in the hemolymph. Furthermore, we identified PDF as a ligand for BNGR-B2. PDF stimulated ecdysone biosynthesis in the PGs, but the stimulation was only observed in the PGs during a specific larval stage. PDF did not affect the transcript level of known ecdysone biosynthetic enzymes, and inhibiting transcription did not suppress ecdysone biosynthesis, suggesting that the effects of PDF might be mediated by translational regulation and/or post-translational modification. In addition, the participation of protein kinase A (PKA), phosphatidylinositol 3-kinase (PI3K), target of rapamycin (TOR) and eukaryotic translation initiation factor 4E (eIF4E)-binding protein (4E-BP) in the PDF signaling pathway was discovered.

## Introduction

Ecdysone is the key regulator of insect growth and development. Ecdysone is synthesized in the PGs and the biosynthesis is intricately regulated by several neuropeptides. To date, four prothoracicotropic peptides (PTTH, FXPRL-amides, insulin and orcokinins) and three prothoracicostatic peptides (PTSP, Bommo-myosuppressin and Bommo-FMRF-amides) have been identified in *Bombyx mori* [1]. The participation of four cytochrome P450 (CYP) enzymes (CYP307A1, Spook (Spo); CYP306A1, Phantom (Phm); CYP302A1, Disembodied (Dib); CYP315A1, Shadow (Sad)); an oxygenase-like protein, Neverland (Nvd); and a short-chain dehydrogenase/reductase, Non-molting glossy/Shroud (Nm-g/Sro), have been reported in the ecdysone biosynthetic pathway [2]–[7]. The transcript levels of *spo*, *dib* and *phm* are upregulated by PTTH and suppressed by Bommo-FMRFamides [8].

All the regulatory mechanisms identified to date were initiated by the discovery of the ligand, after which the receptor and corresponding signaling pathway were investigated. To identify a novel regulatory pathway regulating ecdysone biosynthesis, we focused on the receptors expressed predominantly in the PGs of *B. mori*. Candidate receptor genes were screened by transcriptome analysis using next-generation sequencing (NGS) and subsequent analyses, and we identified the BNGR-B2 as a candidate. *BNGR-B2* was one of the genes identified by the global analysis of neuropeptide GPCR genes in *B. mori* [9], but its ligand and function have not been fully investigated. Here, we show that a neuropeptide, PDF, is a ligand for BNGR-B2 and stimulates ecdysone biosynthesis in the PGs of *B. mori*. PDF is homologous to pigment dispersing hormone (PDH) in Crustacea and has been identified or predicted in insects and nematodes [10]. All the identified insect PDFs and crustacean PDHs consist of octadecapeptides with amidated C-termini, whereas nematode PDFs consist of 20–22 amino acids. In Crustacea, PDH regulates the color change of shielding pigments in the compound eye and the epithelial chromatophoral pigment [11]. Because insects lack chromatophores, PDF was predicted to have a different function in insects, although for many years its function remained unknown. Recently, several functions for PDF have been reported, such as involvement in circadian clock regulation [12], geotaxis [13] and reproduction [14]. In addition, the PDF receptor (PDFR) was identified [15], [16]. Here, we show a novel function for PDF, the stimulation of ecdysone biosynthesis.

## Materials and Methods

### Animals

Two strains of the silkworm *B. mori* were used for this study. The p50T strain was used for NGS analysis and tissue distribution analysis, and the Kinshu x Showa strain was used for other experiments. The p50T strain was reared on mulberry leaves at room temperature. The Kinshu x Showa strain was reared on an artificial diet (SilkMate PS: Nihon Nosan Kogyo) at 25°C under a 16L:8D photoperiod. The first feeding day was designated as day 0.

### Tissue culture and small molecule inhibitors

Dissected PGs were rinsed with Grace's insect culture medium (Gibco) and pre-cultured individually in 100 µl medium in 96-well plates (BD Falcon) at 25°C for 30 min. To evaluate the effect of extracellular Ca^2+^ on ecdysone biosynthesis, lepidopteran saline [17] and lepidopteran saline using NaCl as a replacement for CaCl_2_ were used. For the pharmacological analysis, the PG was pre-treated with the inhibitor for 15 min during the pre-culture period. Following this treatment, the PG was cultured in the desired conditions for the desired period (Ecdysone assay: 3 hours; cAMP assay: 30 min; and Protein phosphorylation assay: 15 min). Actinomycin D, H-89 and cycloheximide (CHX) were purchased from Sigma; Rapamycin was purchased from Santa Cruz Biotechnology; and LY294002 was purchased from Merck. H-89 was dissolved in water. The other inhibitors were dissolved in DMSO. A 1% volume of the appropriate inhibitor was added to the culture medium.

### Transcriptome analysis

Total RNA was isolated from the PGs and brains (partially with corpora cardiaca and corpora allata) of the *Bombyx* larvae p50T strain at the wandering stage using TRIzol (Invitrogen) according to the manufacturer's instructions. Construction of RNA-Seq library was performed as previously described [18], using TrueSeq mRNA Sample Preparation Kit (Illumina). For sequencing, Illumina adaptors were ligated to the cDNA ends, following manufacturers' instructions. Sequence was read on the Genome Analyzer IIx platform by thirty-six base-pair single-end-read. RNA-Seq tags that were mapped to the reference genome sequences and the tags which were mapped without any mismatches were used. Then, RNA-Seq tags were aligned to model transcripts according to the *B. mori* genome annotations to estimate expression levels. The number of RNA-Seq tags aligned to the gene was counted, and the reads per kilobase exon model per million mapped reads (rpkm) was calculated. The expression ratio of the genes was determined by comparing the rpkm in the PGs to the rpkm in the brain. The sequencing data was deposited in DDBJ Sequence Read Archive (Accession number: DRA002282).

### cDNA preparation and gene expression analysis

Total RNA was isolated using the High Pure RNA Tissue Kit (Roche) according to the manufacturer's protocol. The extracted RNA was reverse transcribed with oligo-dT_18_ and Superscript III reverse transcriptase (Invitrogen). The tissue distribution of target genes was evaluated with RT-PCR using GoTaq (Promega), separated on an agarose gel and visualized with ethidium bromide. Quantitative RT-PCR (Q-PCR) was performed on a Thermal Cycler Dice Real Time System using SYBR Premix ExTaq II (Takara Bio). *Ribosomal protein L3* (*RpL3*) was used as an internal standard. The oligonucleotide primers used for the analysis are shown in Table S1.

### Phylogenetic analysis of BNGR-B2

The deduced amino acid sequence of *BNGR-B2* was aligned with the homologous protein from *Aedes aegypti* (GenBank accession number: XP_001653651), *Anopheles gambiae* (XP_313426), *Drosophila melanogaster* (NP_570007), *Tribolium castaneum* (XP_971738), *Apis mellifera* (XP_395896), *Daphnia pulex* (EFX90264), *Marsupenaeus japonicus* (BAH85843) and *Caenorhabditis elegans* (NP_001021172). The calcitonin receptor from *Mus musculus* (AAK56132) was used as an outgroup. All of the sequences were aligned using MUSCLE [19], and the regions used for phylogenetic analysis were selected by Gblocks 0.91b [20]. A phylogenetic tree was generated using ClustalX (Neighbor-Joining method with a bootstrap analysis of 1000 trials).

### HEK293 transfection and cAMP quantification

HEK293 cells were transfected with an empty mammalian expression vector (pME18S) or the full-length BNGR-B2 ORF inserted into the pME18S vector (BNGR-B2/pME18S) using Lipofectamine LTX with PLUS reagent (Invitrogen) according to the manufacturer's instructions. The transfected cells and dissected PGs were treated with a ligand in the presence of 0.5 mM 3-isobutyl-1-methylxanthine. The cAMP assay was performed using the cAMP-Screen Chemiluminescent Immunoassay System (Applied Biosystems) according to the manufacturer's instructions. To extract cAMP from the PG, 100 µl of acidic ethanol (0.1% 10 N HCl, v/v) was added to a gland and vortexed, and then the supernatant was evaporated. The luminescence was measured with a Wallac ARVO SX 1420 Multilabel Counter (PerkinElmer).

### Ecdysteroids extraction and liquid chromatography-tandem mass spectrometry (LC-MS/MS) analysis

Prior to ecdysteroids extraction, the 100 µl of cultured medium was diluted with equal volume of MilliQ water. After that, 400 µl of 1-butanol was added to the sample, vortexed for 3 min and centrifuged at 1,000×*g* for 10 min at 4°C. The 1-butanol phase was transferred to a new tube, and the solvent was evaporated using a centrifugal concentrator. The dried samples were re-dissolved in 50 µl of pure methanol for the following analysis. Quantification of ecdysteroids using LC-MS/MS was performed as previously described [21] with some modifications. Ecdysteroids were separated by reverse-phase HPLC using a PEGASIL C8 column (3 µm, 2×100 mm, Senshu-pak, Senshu-kagaku) with gradient elution of acetonitrile (ACN)/water at a flow rate of 0.2 mL min^−1^ (0–3 min (ACN = 10–75%), 3–20 min (75–85%), 20–25 min (100%)), on a Prominence UFLC system (Shimadzu). The separated ecdysteroids were quantified with the QTRAP5500 MS/MS system (AB SCIEX) using MRM mode. For the quantitative analysis, standard curves were generated with concentrations of 0.98–1000 ng/ml.

### Western blot analysis

Dissected PGs were pre-cultured in Grace's culture medium for 30 min, then cultured with or without PDF or PTTH for 15 min. The cultured PGs were sonicated in 50 mM Tris-HCl (pH 6.8) with protease inhibitor cocktail (Complete, Roche) and phosphatase inhibitor cocktail (Wako), lysed in 1x SDS sample buffer excluding 2-mercaptoethanol (2ME) and bromophenol blue (BPB), and the protein concentration was measured using the BCA protein assay kit (Pierce). After adding 2ME and BPB, 10 µg of protein was loaded in each well and separated on a 12% SDS-PAGE gel. Anti-phospho-ERK antibody, anti-phospho-4E-BP1 antibody, anti-rabbit IgG horseradish peroxidase (HRP)-linked antibody, and anti-mouse IgG HRP-linked antibody were purchased from Cell Signaling Technology (cat #9101, #9459, #7074, #7076), and the α-tubulin antibody was purchased from Sigma-Aldrich (cat #T9026). Immunostar LD (Wako) was used for signal detection.

## Results

### Screening of the receptor responsible for ecdysone biosynthesis

Fifty million 36-bp single-end reads were examined using NGS, and 25 million RNA-Seq tags were uniquely aligned to the *Bombyx* genome sequence. The number of RNA-Seq tags aligned to the gene was counted, and the rpkm was calculated. The rpkm value was used to evaluate the expression level of each gene, and the rpkm in the PGs was compared to the rpkm in the brain to determine an expression ratio. To identify receptor genes that are preferentially expressed in the PGs, the screen was performed using the following criteria: i) the number of RNA-Seq tags was >3,000 and the expression ratio was >10 or ii) the expression ratio was >50. Using these criteria, *torso* and two orphan GPCR genes (*BNGR-A34* and *BNGR-B2*) were selected from the 21,302 genes estimated to be expressed in the PGs. Because *torso* is a known PTTH receptor gene and the expression of *BNGR-A34* was not confirmed in PGs, these were excluded as candidate genes. Thus, BNGR-B2 was selected as the candidate.

### Characterization of BNGR-B2

**Figure 1:**
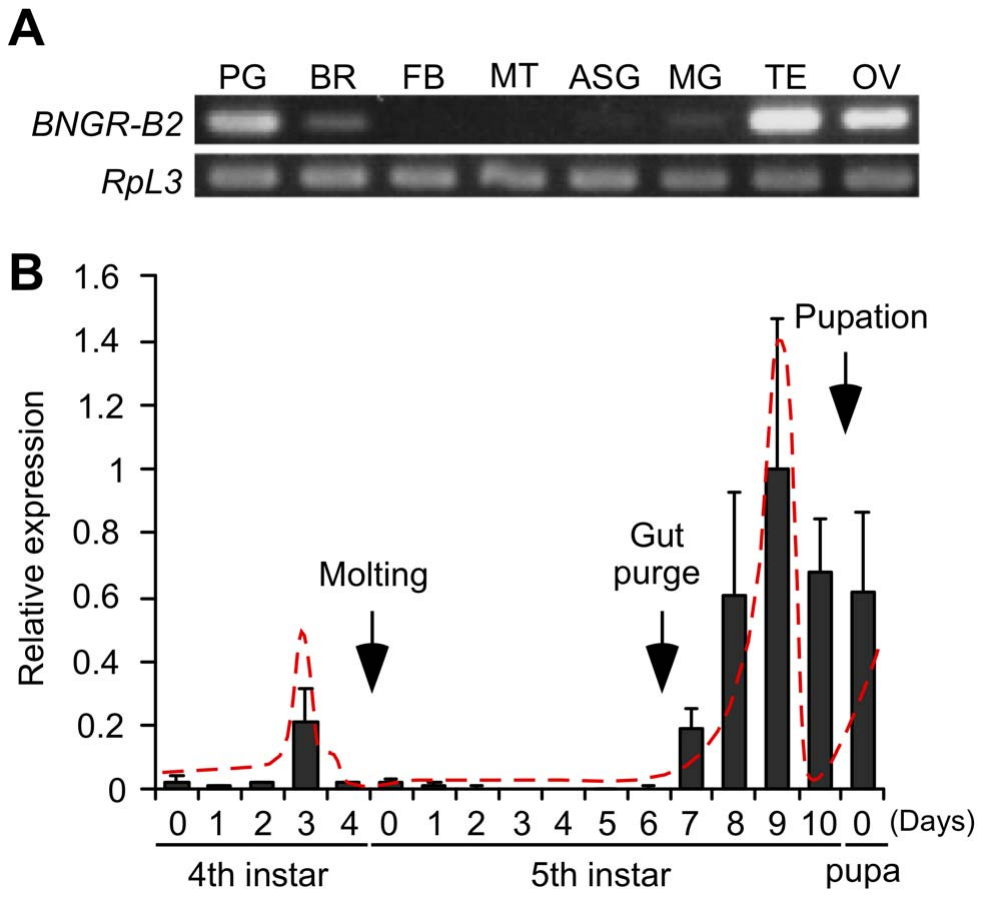
Screening of candidate receptors. (A) Tissue distribution of *BNGR-B2*. The expression of *BNGR-B2* was measured by RT-PCR in the selected tissues from gut-purged fifth instar larvae (p50T strain). PG: prothoracic gland, BR: brain, FB: fat body, MT: Malpighian tubule, ASG: anterior silk gland, MG: midgut, TE: testis and OV: ovary. (B) Developmental profile of *BNGR-B2* in the PGs. The expression of *BNGR-B2* was measured by Q-PCR. The timing of molting, gut purge and pupation in our rearing conditions is indicated with arrows. Each datum point represents the mean ±SEM (n = 3). The dashed line indicates the outline of the hemolymph ecdysteroid titer described by Koyama et al., 2004 (4th instar), Sakurai et al., 1998 (5th instar) and Kaneko et al., 2006 (5th instar). (A, B) *RpL3* was used as an internal standard.

The tissue distribution of *BNGR-B2* was investigated in the gut-purged fifth instar larvae using RT-PCR. As shown in Figure 1A, *BNGR-B2* was predominantly expressed in the PGs and gonads (testis and ovary). The expression profile of *BNGR-B2* in the PGs was determined using Q-PCR in fourth and fifth instar larvae and the first day of pupae (Figure 1B). *BNGR-B2* expression peaked on day 3 in fourth instar larvae (IV3). Furthermore, during the larval-pupal metamorphosis, the expression of *BNGR-B2* began to increase on day 7 and peaked on day 9 in fifth instar larvae (V7 and V9), after which the expression decreased. Thus, the expression profile of *BNGR-B2* in the PGs correlated with the ecdysteroid titer in the hemolymph [22]–[24].

**Figure 2:**
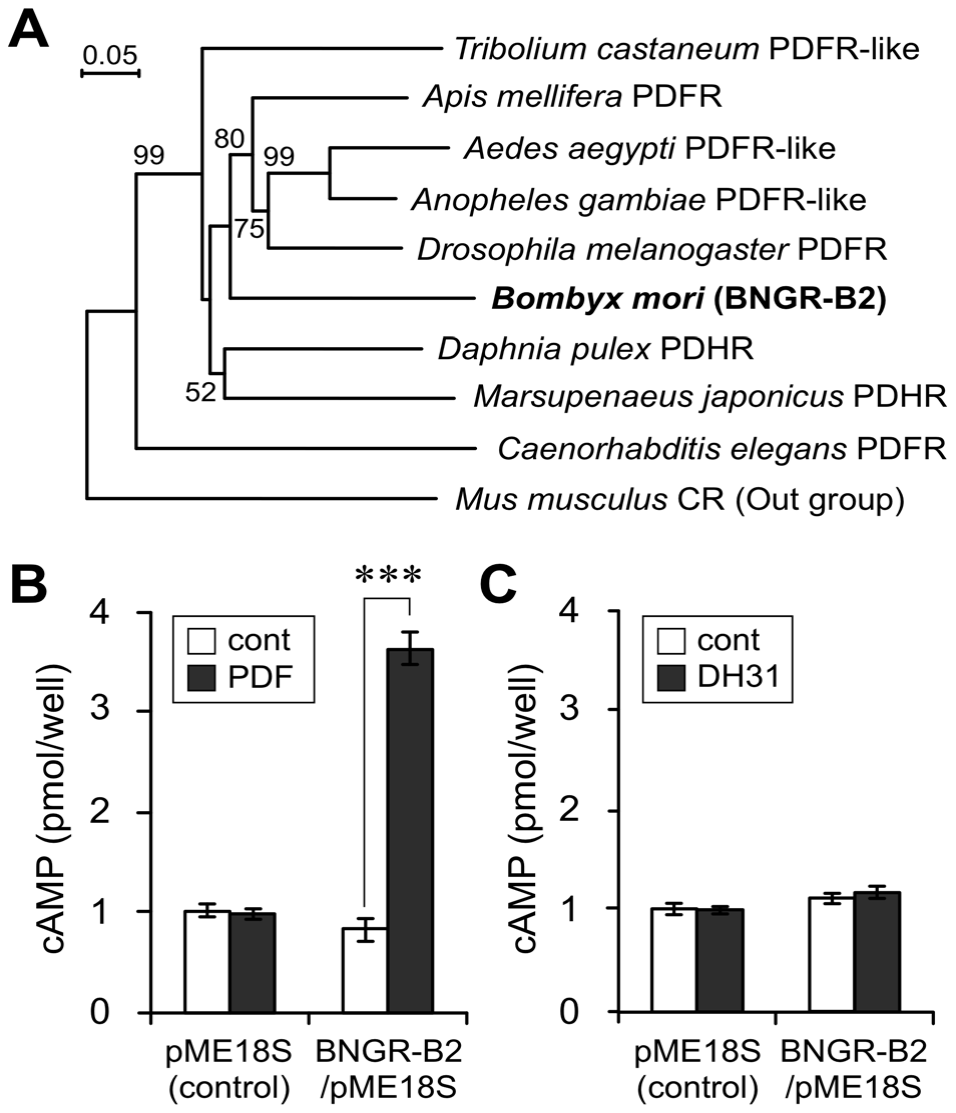
Characterization of BNGR-B2. (A) Phylogenetic relationship of BNGR-B2 and highly homologous receptors. The tree was generated based on the amino acid sequences of selected regions with the neighbor-joining method using the ClustalX multiple alignment program and a bootstrap value of 1000 trials for each branch position. The indicated numbers are the bootstrap values as a percentage of 1000 replicates, and the scale bar indicates 0.05 changes per residue. Bootstrap values greater than 50% are indicated. The *Mus musculus* calcitonin receptor (CR) was used as an outgroup. (B) Ligand-binding analysis of BNGR-B2 by examining the change in intracellular cAMP levels. BNGR-B2-expressing HEK293 cells were treated with 1 µM of the candidate BNGR-B2 ligands (PDF and DH31). Each datum point represents the mean ±SEM (n = 5). Statistically significant differences were evaluated by Student's *t*-test (***P<0.001).

The deduced amino acid sequence of BNGR-B2 was highly similar to the PDFR in other insect species and nematodes and to the pigment-dispersing hormone receptor (PDHR) in Crustacea (Figure 2A). In *Drosophila*, PDF and diuretic hormone 31 (DH31) have been reported as PDFR ligands [25]; therefore, *Bombyx* PDF and DH31 were selected as candidate ligands for BNGR-B2. A heterologous expression system was employed for ligand screening [26]. HEK293 cells were transfected with the BNGR-B2/pME18S vector, and binding of the candidate ligands was evaluated by changes to the intracellular cAMP level. PDF only stimulated an increase in the intracellular cAMP level in BNGR-B2-transfected cells (Figure 2B), whereas DH31 had no effect (Figure 2C).

### Effect of PDF and DH31 on ecdysone biosynthesis in the PGs

**Figure 3:**
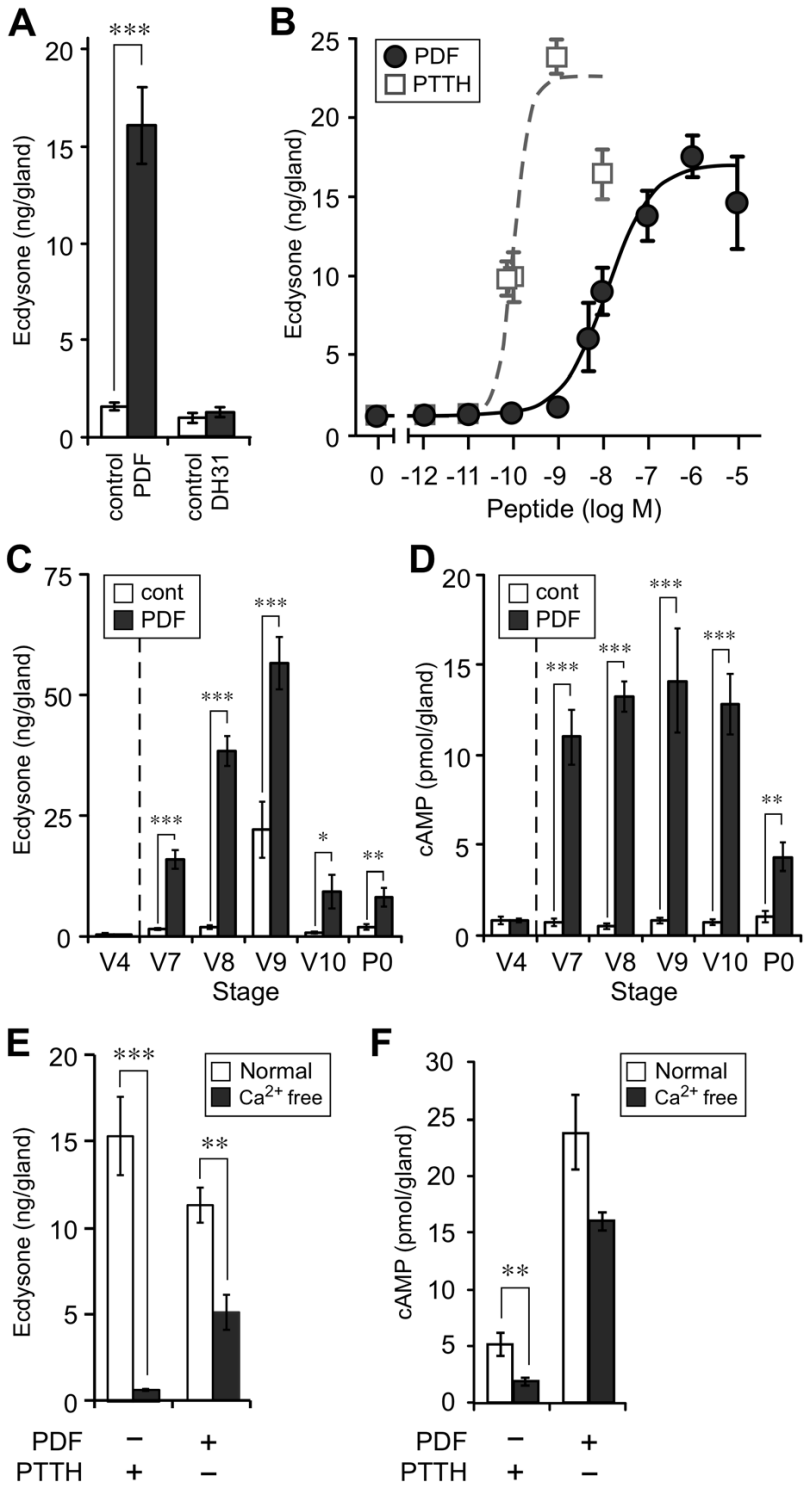
Prothoracicotropic activities of PDF *in vitro*. (A) Effect of PDF (1 µM) and DH31 (1 µM) on ecdysone biosynthesis in the PGs of V7 larvae (n = 14 and 12). (B) Dose-response curves for PDF and PTTH on ecdysone biosynthesis in the PGs of V7 larvae. Closed circles indicate PDF (n = 4–24) and open squares indicate PTTH (n = 10–38). (C, D) Developmental changes in PDF responsiveness on (C) ecdysone biosynthesis (n = 8–14) and (D) intracellular cAMP level (n = 5–6). (E) Effect of extracellular Ca^2+^ on ecdysone biosynthesis (n = 6). (F) Effect of extracellular Ca^2+^ on the change in intracellular cAMP levels (n = 4–8). Statistically significant differences were evaluated by Student's *t*-test (***P<0.001, **P<0.01, *P<0.05).

The effect of PDF and DH31 on ecdysone biosynthesis was evaluated in the PGs of *B. mori*. The PGs of V7 larvae were cultured with PDF or DH31, and the amount of ecdysone synthesized was measured using LC-MS/MS [21]. In support of the ligand screening results (Figure 2B and C), only PDF stimulated ecdysone biosynthesis in the PGs (Figure 3A). The dose-response to PDF in cultured PGs of V7 larvae was investigated using the amount of ecdysone synthesized and compared to the PTTH dose-response. PDF showed the highest prothoracicotropic activity at concentrations of 10^−6^ M, and the EC_50_ was 12.8 nM (Figure 3B). On the other hand, PTTH showed the highest prothoracicotropic activity at concentrations of 10^−9^ M, and the EC_50_ was 107.6 pM (Figure 3B).

The PGs from several different stages were cultured with or without PDF, and the amount of ecdysone synthesized was measured (Figure 3C). PDF stimulated ecdysone biosynthesis in the PGs of post-gut-purged larvae and pupae (V7-P0) but not in the PGs of fifth instar day 4 larvae (V4). These results are consistent with the expression level of *BNGR-B2*, which was very low in the PGs of V4 larvae (Figure 1B). The amount of ecdysone synthesized began to increase from V7 and peaked at V9, after which it dramatically decreased. Basal ecdysone biosynthesis in the cultured PGs also peaked at V9. Furthermore, the changes in intracellular cAMP levels were only observed in the PGs derived from post-gut-purged larvae and pupae (Figure 3D). Accordingly, the PDF signal is transmitted into the cells via BNGR-B2, and cAMP is used as a second messenger.

The importance of extracellular Ca^2+^ to PTTH-induced ecdysone biosynthesis has been previously reported. Therefore, we evaluated the effect of extracellular Ca^2+^ depletion on PDF-induced ecdysone biosynthesis using Ca^2+^-free lepidopteran saline. Without extracellular Ca^2+^, PTTH did not induce ecdysone biosynthesis, as reported previously (Figure 3E) [27]. In contrast, PDF stimulated ecdysone biosynthesis without extracellular Ca^2+^, although the amount of ecdysone synthesized was approximately 50% of that produced in normal Ca^2+^-containing lepidopteran saline (Figure 3E). In addition, Ca^2+^ depletion in the culture medium suppressed PTTH-induced upregulation of intracellular cAMP levels, whereas PDF-induced cAMP upregulation was not affected (Figure 3D and F).

### Signaling pathway of PDF-mediated ecdysone biosynthesis

**Figure 4:**
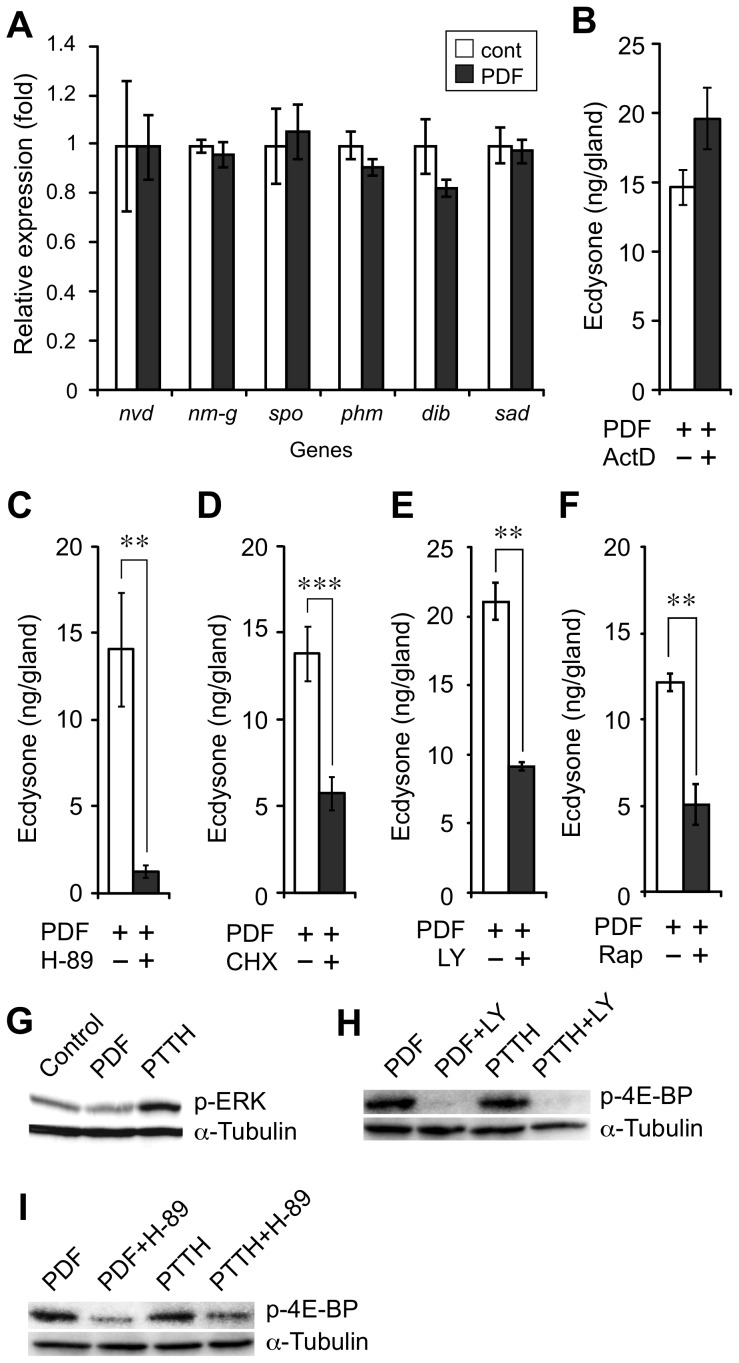
Signaling pathways involved in PDF-induced ecdysone biosynthesis. (A) Effect of PDF on the transcript level of ecdysone biosynthesis-related enzymes in the PGs. PGs of V7 larvae were treated with or without PDF. The transcript levels of *nvd*, *nm-g*, *spo*, *phm*, *dib* and *sad* were quantified with Q-PCR. Each datum point represents the mean ±SEM (n = 3). (B-D) Effect of (B) transcript inhibitor (actinomycin D: ActD, 10 µM), (C) PKA inhibitor (H-89, 0.1 mM) and (D) translation inhibitor (cycloheximide: CHX, 0.2 mM) on PDF-induced ecdysone biosynthesis. Each datum point represents the mean ±SEM (n = 10). (E, F) Effect of (E) PI3K inhibitor (LY294002: LY, 50 µM) and (F) TOR inhibitor (rapamycin: Rap, 10 µM) on PDF-induced ecdysone biosynthesis. Each datum point represents the mean ±SEM (n = 3 and 4). Statistically significant differences were evaluated by Student's *t*-test (***P<0.001, **P<0.01). (G) Effect of PDF on the levels of p-ERK and p-4E-BP in cultured PGs. The phosphorylated proteins were examined by immunoblotting, and α-tubulin was used as a loading control. (H, I) Effect of (H) PI3K inhibitor (LY294002: LY, 50 µM) and (I) PKA inhibitor (H-89, 0.1 mM) on p-4E-BP levels in cultured PGs. The phosphorylated proteins were examined by immunoblotting, and α-tubulin was used as a loading control.

Because PDF stimulated the increase in intracellular cAMP levels in the PGs, the activation of PKA is likely involved in the PDF signaling pathway. One of the pathways downstream of active PKA is cAMP response element-binding protein (CREB)-mediated transcriptional regulation. First, we evaluated the effect of PDF on the transcript level of known ecdysone biosynthetic enzymes (Nvd, Nm-g, Spo, Phm, Dib and Sad) using Q-PCR. PDF did not affect the transcript levels of any of the selected genes (Figure 4A). Furthermore, pharmacological analysis using the transcription inhibitor (actinomycin D) did not suppress PDF-induced ecdysone biosynthesis, confirming the Q-PCR results (Figure 4B). Therefore, *de novo* transcription was not required for PDF-mediated ecdysone biosynthesis for at least 3 hours. On the other hand, a PKA inhibitor (H-89) and a translation inhibitor (CHX) clearly inhibited ecdysone biosynthesis (Figure 4C and D). Accordingly, *de novo* protein synthesis and/or post-translational modification of protein(s) such as phosphorylation might be important for regulating ecdysone biosynthesis.

### PDF-mediated protein phosphorylation

During PTTH-induced ecdysone biosynthesis, the participation of the mitogen-activated protein kinase (MAPK) and TOR pathways has been reported [28], [29]. To investigate the difference between the PDF- and the PTTH-induced ecdysone biosynthetic pathways, the phosphorylation status of proteins in the MAPK and TOR pathways was investigated. Phosphorylated extracellular signal-regulated kinase (p-ERK) and phosphorylated 4E-BP (p-4E-BP) were used to evaluate the participation of the MAPK and TOR pathways, respectively. The level of p-ERK was upregulated when the PGs were treated with PTTH but not when the PGs were treated with PDF (Figure 4G). In contrast to p-ERK, the level of p-4E-BP was upregulated by both PDF and PTTH (Figure 4G). Because PI3K-mediated phosphorylation of 4E-BP has been reported during PTTH-induced ecdysone biosynthesis [29], the effect of a PI3K inhibitor (LY294002) on the phosphorylation of 4E-BP by PDF was investigated. The PI3K inhibitor clearly suppressed the ecdysone biosynthesis and phosphorylation of 4E-BP induced by PDF and PTTH (Figure 4E and H). Furthermore, a PKA inhibitor suppressed both the PDF- and PTTH-induced phosphorylation of 4E-BP (Figure 4I). These results suggest that the TOR-mediated phosphorylation of 4E-BP is regulated by the PKA-PI3K pathway.

## Discussion

PDF is primarily considered an important component for regulating circadian rhythms in insects, but here we showed that PDF has a novel function in the stimulation of ecdysone biosynthesis. In many cases, a single hormone has multiple functions regulating many physiological phenomena. Thus, both the regulation of receptor expression in the target tissue and the regulation of hormone release are important for the hormone to only affect the target tissue at the appropriate time. In the case of PTTH, the titer of the hormone in the hemolymph is clearly correlated with the ecdysteroid titer [30], whereas the gene expression pattern of the PTTH receptor Torso is not [31]. However, the PGs of V4 (a low ecdysone level period) are capable of responding to PTTH stimulus and synthesize ecdysone *in vitro* [8]. Thus, the effect of PTTH is predominantly regulated by the timing of hormone secretion. By contrast, the gene expression of BNGR-B2 (PDFR) was clearly correlated with the hemolymph ecdysteroid titer (Figure 1B), and the amount of ecdysone synthesized and the increase in intracellular cAMP levels induced by PDF was clearly correlated with the expression level of *BNGR-B2* (Figure 3C and D). Therefore, the effect of PDF can be regulated by the expression level of the receptor and/or the timing of hormone secretion. When these results are examined as a whole, PDF plays important roles in regulating the timing of ecdysone biosynthesis.

In insects, neuropeptide hormones affect the target tissue via humoral and neuronal pathways. The EC_50_ value of the humoral pathway-mediated tropic hormone, PTTH, was approximately 108 pM, whereas the neuronal pathway-mediated tropic hormones, diapause hormone and orcokinins, were 270 nM and 12.6–46.8 nM, respectively [32], [33]. Because the EC_50_ value of PDF was 12.8 nM, PDF appears to affect PGs via the neuronal pathway, although further research into this topic is required.

The localization of PDF has been well studied in *Drosophila* and *Locusta*, but little is known in *Bombyx*. The localization of PDF in a subset of clock neurons in the brain and in abdominal PDF neurons in the abdominal ganglia has been reported in several insect species [10], [34]. PDF from the brain is secreted in a circadian manner from a subset of clock neurons, and it modulates the length of periodicity. However, the secretion of PDF into the hemolymph from the abdominal ganglia has been reported in the larvae of *Locusta migratoria*. Thus, hormonal functions for PDF have been suggested, although the function remains unknown [35]. We investigated the transcript level of PDF in the central nervous system (CNS) of *Bombyx* larvae, and the results were consistent with previous reports examining other species. PDF was predominantly expressed in the brain and abdominal ganglia (Figure S1). Thus, the possibility remains that PDF affects PGs via the humoral pathway.

PDF or PDFR null mutant flies show an aberrant behavioral circadian rhythmicity and severe negative geotaxis phenotype [25], [36]. On the other hand, little is known about the effect of PDF depletion on larval development. Given the effect of PDF on ecdysone biosynthesis, PDF might have important function(s) in the regulation of larval development, including the circadian secretion of ecdysone.

Extracellular Ca^2+^ and an increase in cAMP levels are important for PTTH-induced ecdysone biosynthesis. PTTH cannot stimulate ecdysone biosynthesis when extracellular Ca^2+^ is depleted, whereas a cell-permeable cAMP analog, dibutyryl cyclic AMP (dbcAMP), can induce ecdysone biosynthesis *in vitro*. The increase in intracellular cAMP is thought to be regulated by Ca^2+^ signaling in the PTTH signaling pathway [27], [37]. Our results also clearly showed that extracellular Ca^2+^ is important for the ecdysone biosynthesis and intracellular cAMP increase induced by PTTH (Figure 3E and F). PDF significantly upregulates the intracellular cAMP level in PGs; therefore, cAMP is also used as a second messenger in the PDF signaling pathway. Both PDF and PTTH stimulate the increase in intracellular cAMP levels, although the degree of increase was different between PDF and PTTH. The intracellular cAMP increase induced by PDF was higher than that induced by PTTH (Figure 3F), whereas the amount of synthesized ecdysone was lower than that induced by PTTH (Figure 3B and E). Accordingly, non-cAMP-mediated signaling pathways are also involved in the PDF-mediated ecdysone biosynthetic pathway. PDF was capable of stimulating ecdysone biosynthesis when extracellular Ca^2+^ was depleted (Figure 3E). However, the amount of ecdysone synthesized was decreased by nearly 50%. Therefore, extracellular Ca^2+^ is not essential for inducing ecdysone biosynthesis with PDF but is required for the full induction of ecdysone biosynthesis. Because extracellular Ca^2+^ depletion did not suppress the upregulation of intracellular cAMP levels induced by PDF (Figure 3F), the extracellular Ca^2+^ might affect ecdysone biosynthesis via other pathway(s); however, the details remain unclear. Altogether, these results indicate that PDF-induced ecdysone biosynthesis might be regulated independently by both cAMP- and extracellular Ca^2+^-mediated pathways.

Because PDF increased the intracellular cAMP level in PGs, the involvement of PKA in the PDF signaling pathway was expected. Indeed, the PKA inhibitor H-89 inhibited PDF-induced ecdysone biosynthesis (Figure 3C). Three main possibilities were suggested to occur downstream of PKA activation: PKA-mediated phosphorylation of protein(s), transcriptional regulation via CREB and translational regulation via 4E-BP. In mammals, most of the CYP enzymes involved in steroid hormone biosynthesis are transcriptionally regulated [38]. In PTTH- and dbcAMP-induced ecdysone biosynthesis, ecdysone biosynthesis was suppressed by a transcription inhibitor [39]. Furthermore, PTTH upregulated the transcript level of some ecdysone biosynthetic enzymes [8]. However, transcription inhibitor did not suppress PDF-induced ecdysone biosynthesis (Figure 4B), and PDF did not affect the transcript level of any of the known ecdysone biosynthetic enzymes examined (Figure 4A). Thus, the pathways regulating PDF- and PTTH-induced ecdysone biosynthesis appear to be different.

A translation inhibitor significantly suppressed PDF-induced ecdysone biosynthesis (Figure 4D), suggesting that *de novo* protein synthesis is essential for PDF-induced ecdysone biosynthesis. This result is consistent with PTTH-induced ecdysone biosynthesis [39]. In the case of PTTH, upregulation of the basal protein synthesis level and also some specific proteins has been reported [40], [41]. Furthermore, PTTH-induced changes to the phosphorylation state of several proteins have been reported [42]. The phosphorylation of ERK, a component of the MAPK pathway, is involved in PTTH signaling and is important for inducing ecdysone biosynthesis in the PGs [43]. However, the MAPK pathway was not involved in PDF signaling. The participation of the PI3K-TOR-4E-BP pathway has also been reported during PTTH-induced ecdysone biosynthesis [29], and the pathway participates in the PDF signaling pathway (Figure 4E, F and H). In addition, because a PKA inhibitor suppressed the ecdysone biosynthesis and phosphorylation of 4E-BP (Figure 4C and I), PKA is upstream of the PI3K-TOR-4E-BP pathway. Therefore, the initial signaling pathway is different between PDF and PTTH, whereas the PKA-PI3K-TOR-4E-BP pathway is important for the regulation of ecdysone biosynthesis by both PDF and PTTH.

**Figure 5:**
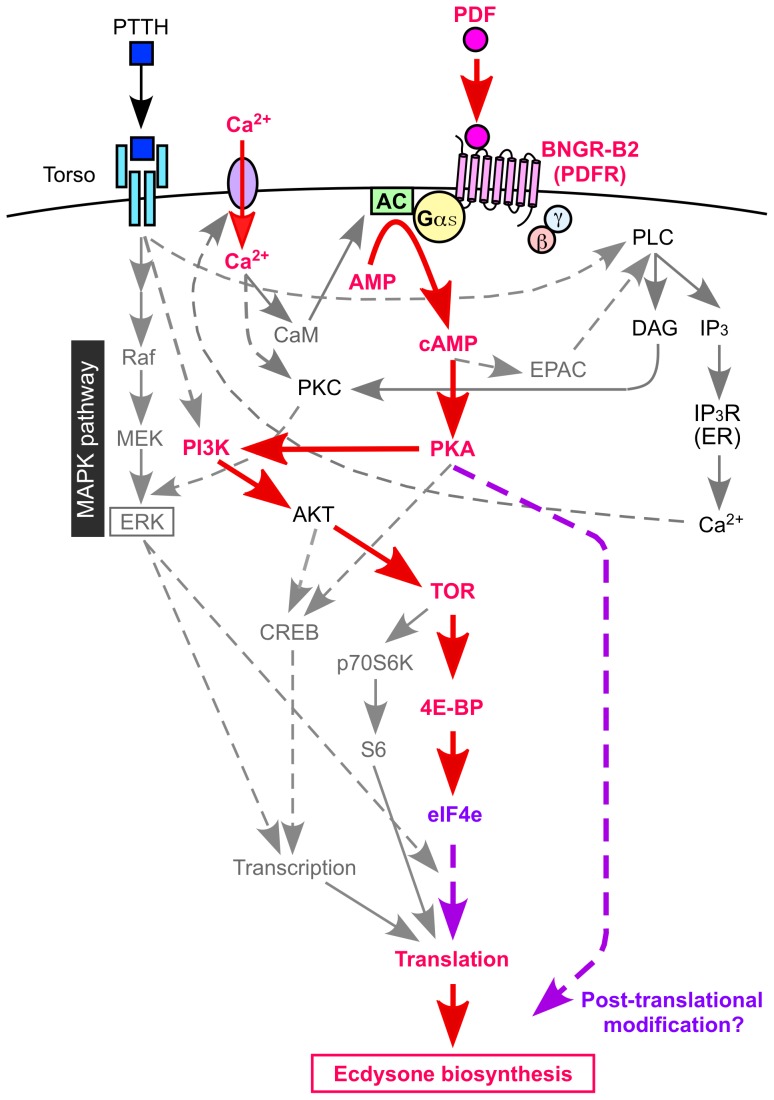
Integration of the PDF signaling model with the known PTTH signaling pathway. Solid lines indicate demonstrated or highly likely pathways, and dashed lines indicate hypothetical pathways. Gαs: G protein αs subunit, AC: adenylate cyclase, AMP: adenosine monophosphate, cAMP: cyclic AMP, EPAC: exchange protein directly activated by cAMP, eIF4e: eukaryotic translation initiation factor 4E, 4E-BP: eIF4E binding protein, TOR: target of rapamycin, PKA: protein kinase A, PKC: protein kinase C, PI3K: phosphatidylinositol 3-kinase, AKT: protein kinase B, CREB: cAMP response element-binding protein, MAPK: mitogen-activated protein kinase, ERK: extracellular signal-regulated kinase, MEK: MAP kinase kinase, Raf: MAP kinase kinase kinase, S6: ribosomal protein S6, p70S6K: 70 kDa S6 kinase, PLC: phospholipase C, DAG: diacylglycerol, IP_3_: inositol 1,4,5-trisphosphate, IP_3_R: IP_3_ receptor, CaM: calmodulin.

Figure 5 shows the PDF-induced ecdysone biosynthesis signaling model with the known PTTH signaling. PDF binds to BNGR-B2 and upregulates intracellular cAMP levels. cAMP activates PKA, which directly or indirectly affects the PI3K-TOR-4E-BP pathway. Phosphorylated 4E-BP might be released from eIF4e, and translation is initiated. Furthermore, post-translational modification by PKA and/or other kinase(s) might play an important role in regulating ecdysone biosynthesis. In addition to the cAMP-mediated pathway, PDF regulates ecdysone biosynthesis via an extracellular Ca^2+^-mediated pathway. Thus, PDF regulates ecdysone biosynthesis via two different pathways, partially overlapping with the PTTH signaling pathway. These findings aid in the elucidation of the intricate neuropeptide network regulating ecdysone biosynthesis.

## Supporting Information

Figure S1 **Gene expression of PDF in the CNS.** Expression of *PDF* in the CNS was evaluated using standard RT-PCR. BR: brain; SOG: suboesophageal ganglion; TG1-3: thoracic ganglion 1-3; and AG1-8: abdominal ganglion 1-8. *RpL3* was used as an internal standard.
(TIFF)

Table S1 **Oligonucleotide primers used for PCR.**
(DOCX)
