# Supplementary figures and images for: Pigment Dispersing Factor Regulates Ecdysone Biosynthesis via Bombyx Neuropeptide G Protein Coupled Receptor-B2 in the Prothoracic Glands of Bombyx mori
Source: PLoS One. 2014 Jul 29;9(7):e103239. doi: 10.1371/journal.pone.0103239 (PMC4114559; doi:10.1371/journal.pone.0103239)

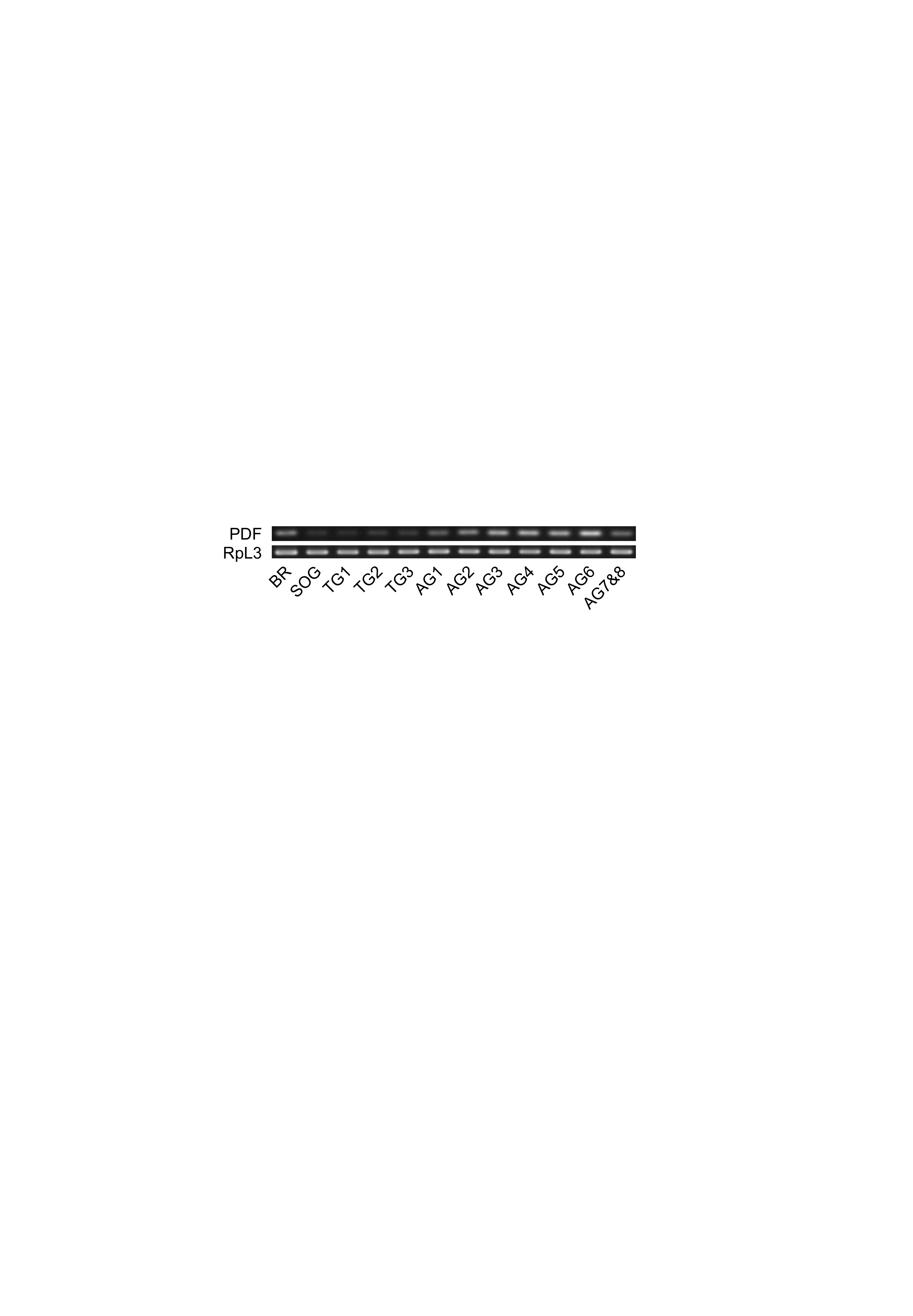

Supplement: Figure S1 — Gene expression of PDF in the CNS. Expression of PDF in the CNS was evaluated using standard RT-PCR. BR: brain; SOG: suboesophageal ganglion; TG1-3: thoracic ganglion 1-3; and AG1-8: abdominal ganglion 1-8. RpL3 was used as an internal standard. (TIFF) [file pone.0103239.s001.tiff]
