# Supplementary material for: Pigment Dispersing Factor Regulates Ecdysone Biosynthesis via Bombyx Neuropeptide G Protein Coupled Receptor-B2 in the Prothoracic Glands of Bombyx mori
Source: PLoS One. 2014 Jul 29;9(7):e103239. doi: 10.1371/journal.pone.0103239 (PMC4114559; doi:10.1371/journal.pone.0103239)
Supplement: Table S1 — Oligonucleotide primers used for PCR. (DOCX) [file pone.0103239.s002.docx]

**Table S1.** Oligonucleotide primers used for PCR

| RT-PCR |  |  |  |  |
| --- | --- | --- | --- | --- |
| Gene | Accession no. | Forward primer (5'-3') | Reverse primer (5'-3') | Ref. |
| *BNGR-B2* | AB330458 | GGCTTCTTCATCGCGCTAAT | ACCATGTGCAATTCGAGACG |  |
| *PDF* | AB298933 | GGAATCTTCCTACGGATCTGATG | GCCTTGATGGATGGTCTAACG |  |
| *RpL3* | AB024901 | AGCACCCCGTCATGGGTCTA | TGCGTCCAAGCTCATCCTGC | *1 |
|  |  |  |  |  |
| Q-PCR |  |  |  |  |
| Gene | Accession no. | Forward primer (5'-3') | Reverse primer (5'-3') |  |
| *BNGR-B2* | AB330458 | GTGCAATGCTCGTCCAAGTG | TTCCTCGGGTCATCGTAGTGT |  |
| *spook* | AB206555 | CTACACGAAGACCCGACCAT | GCGTCGATTTCCTGACGTAT |  |
| *phantom* | AF484413 | TTATGGCAGCAATCTGTGAAACTC | TTCTGGTTCTTCCCAAACATTAGG | *2 |
| *disembodied* | AB198340 | AGGTTGCCGCAGTTTATACG | GAAACGGATGAATGGTCTCG |  |
| *shadow* | AB167737 | TTTGGGACCTTATTTGCTCG | TTGATCGTTCCTGTCCCATC |  |
| *neverland* | AB232986 | TCTTCGAACACGGCGTGCCC | ACAAAATCGCGGAGAACGCAAAC |  |
| *nm-g* | AB361434 | TGTGATAGTGGACTCGGTTGGGC | GGCTTTCGCTGCTTCGGTTTC | *3 |
| *RpL3* | AB024901 | TGGCACACAAAGAAGCTACCC | TGACCAGCACGAGCTACAGTG |  |
| *1: Matsuoka T, Fujiwara H (2000) [44]; *2: Warren et al. (2004) [45]; *3: Niwa et al. (2010) [6] | | | |  |
